# Process control: simply a matter of efficiency or of survival and costs? A single-centre quality improvement project in living donor renal transplant

**DOI:** 10.1186/s12913-023-09183-3

**Published:** 2023-02-23

**Authors:** Filippo Paoletti, Vittori Giorgio, Adel Jaser, Natalia Romina Zanoni, Walter Ricciardi, Franco Citterio, Antonio Giulio De Belvis

**Affiliations:** 1 Clinical Pathways and Outcome Evaluation Unit, Fondazione Policlinico Universitario A.Gemelli IRCSS, Rome, Italy; 2 Società Italiana di Ginecologia e Ostetricia, Rome, Italy; 3 Lean Program Unit, IREN, Turin, Italy; 4 Department of Medical and Surgical Sciences, Urology, Nephrology and Renal Transplant Area, Fondazione Policlinico Universitario A.Gemelli IRCSS, Rome, Italy; 5 Section of Hygiene, Department of Health Science and Public Health, Università Cattolica del Sacro Cuore, Rome, Italy

**Keywords:** Lean methodology, Quality improvement, Living donor, Renal transplantation, Clinical governance

## Abstract

**Background:**

Rising incidence and prevalence of end-stage renal disease is a worldwide concern for sustainability of healthcare systems and societies. Living donor renal transplant [LDRT] provides highest health achievements and cost containment than any alternative form of renal replacement therapy. Nonetheless, about 25% of potential LDRTs are missed for causes directly related with inadequate timing in donor assessment. Our quality improvement (QI) project implement process control tools and strategy aiming at reducing total evaluation time for donor candidates and minimizing dialysis exposure for intended recipients, which are the two main determinants of clinical outcomes and costs.

**Methods:**

The study includes patients who underwent donor nephrectomy between January 1, 2017 and December 31, 2021. Six Sigma DMAIC approach was adopted to assess Base Case performance (Jan2017-Jun2019) and to design and implement our QI project. Study of current state analysis focused on distribution of time intervals within the assessment process, analysis of roles and impacts of involved healthcare providers and identification of targets of improvement. Improved Scenario (Jul2019-Dec2021) was assessed in terms of total lead time reduction, total pre-transplantation dialysis exposure and costs reduction, and increase in pre-emptive transplantations. The study was reported following SQUIRE 2.0 Guidelines for QI projects.

**Results:**

Study population includes 63 patients, 37 in Base Case and 26 in Improved Scenario. Total lead time reduced from a median of 293 to 166 days and this in turn reduced pre-transplantation dialysis exposure and costs by 45%. Rate of potential pre-emptive donors’ loss changes from 44% to 27%.

**Conclusions:**

Lean methodology is an effective tool to improve quality and efficiency of healthcare processes, in the interest of patients, healthcare professionals and payers.

## Background

Chronic kidney disease is one of the most important causes of disability worldwide and prevalence and incidence have increased considerably in the last decades, mainly driven by the epidemics of diabetes [1, 2]. Although dialysis is traditionally considered the inevitable fate for patients with end stage renal disease [ESRD], robust evidence encourages expansion of renal transplantation which offers superior outcomes, in terms of survival and quality of life, at considerably lower costs for the healthcare system [3–10]. LDRT, in particular, provides the best chance of long-term survival and quality of life due to prolonged allograft survival and potential to avoid dialysis completely [11–14]. A study conducted by Axelrod DA et al. reveals that at 10 years LDRT increases survival up to 57% and reduces expected expenditures for ESRD therapy by 13% compared with dialysis [15].

Unfortunately, in Italy LDRT represents only 16% of renal transplant activity and both living and deceased donor programmes are characterized by chronic organ shortage. At the end of 2020, the Italian National Transplant Centre reported 6132 waitlisted patients, an average waiting time for a deceased donor transplant of 3,2 years and 2.3% waitlist mortality rate [16]. In this scenario, LDRT represents a strategic asset to decrease limitations in organ supply. Primary concern of any living donor (LD) programme is to advocate for donor safety, to be achieved by adopting an efficient donor assessment pathway. Such efficiency can be defined by three levels of appropriateness: clinical management, donor safety and process duration. Guidelines carefully describe medical, ethical and procedural elements that guarantee completeness and safety in evaluating donor and recipient [17, 18], while indications on appropriate timing are infrequent. Nonetheless, it is well acknowledged that prolonged donor evaluation might extend time on dialysis for waiting recipients or prevent opportunities for pre-emptive transplantations, therefore reducing potential outcomes or increasing costs [8, 11, 19–21]. A scoping review on efficiency of donor evaluation in LDRT programmes claims that, among the examined population, up to one fifth of potential living transplants were lost due to intended recipient death or illness or receipt of a deceased donor organ [22, 23]. In Italy, these same reasons are responsible for up to 25 percent of LDs’ dropout rate [24]. Whether these missed opportunities of LDRT could have been averted by a quicker evaluation process remains only a hypothesis, however this suspect is strong enough to suggest further investigation. As a matter of fact, a QI project conducted in Northern Ireland, implementing a one-day donor assessment model for intended donors, recorded an increase of 80 and 84 percent in the number of LD transplants and pre-emptive transplants, respectively, and the authors believe this quick and donor-friendly pathway gave the main contribution to these achievements [25].

Therefore, we decided to implement Six Sigma lean methodology to appraise current donor assessment at our transplant centre and to implement an interventions in order to shorten evaluation time.

## Methods

### Population, setting and measures

Study population includes living donors aged 18 or more, who referred to our transplant centre between January 1, 2017 and December 31, 2021 and underwent donor nephrectomy in the following months.. Patients who interrupted/suspended evaluation process or whose data were incomplete or irretrievable were excluded. Crossover LDRT programs were also excluded.

We calculated median LD *lead time*, i.e. time from referral until donor nephrectomy*,* and *care time,* i.e. time to conclude each phase of the evaluation process*.* We also calculate *median dialysis time* and *costs* accrued by intended recipients while donor evaluation is underway. To simplify our analysis, we estimated costs as if all recipients on dialysis had undergone haemodialysis three times a week, the most frequent dialysis scheme in Italy.

Data on recipients were also collected and used to further explain the outputs of the intervention, even though they are not directly affected by the study protocol.

### QI strategy

We implemented Lean Methodology and DMAIC approach (Define – Measure – Analyse – Innovate – Control) [26–29]. The *Base Case* cohort includes patients who referred at our centre between January 2017 and June 2019 (30 months). These data were drawn into a Value Stream Map (VSM) to assess sources of delay and poor quality as well cycle and waiting time, here defined as *care time* and *transitional time*, respectively. A Pareto chart and Fishbone analysis was then developed to assess impact of each phase on final output, to investigate causes and to establish intervention priorities. The prospective phase of the study – the *Improved Scenario* – includes patients who referred to our transplant centre between July 2019 and December 2021 (30 months). This period was characterized by a change in healthcare providers’ awareness of critical areas and delays as well as interventions adopted to enhance living donors’ management, such as institution of a dedicated case manager and tracking system. All this effort resulted in a Renal Transplant Fast Track, i.e. a dedicated protocol approved in October 2020 to organize and monitor living donor assessment.

### Outcome

The goal of the study is twofold. Firstly, to appraise living donor assessment process according to DMAIC principles and to unmask delays and potential areas for implementation. Secondly, to adopt targeted interventions able to affect and improve all major determinants of clinical outcomes and healthcare costs, such as pre-transplantation dialysis exposure and pre-emptive status.

### Statistical analysis and reporting

Sample size represents the entire population with few exceptions based on exclusion criteria. Outliers, whose values are more than 1.5 times larger or smaller than Q3 or Q1, respectively, were excluded. Continuous variables are presented as median and IQR. Categorial variables are presented as frequency and percentage. A descriptive analysis of the results is conducted measuring differences between pre and post intervention performances.

The study is reported according to SQUIRE 2.0 reporting guidelines for QI projects [30].

## Results

Table 1 summarizes baseline characteristics of included couples.

**Table 1:** Population characteristics

	**Base Case** Jan17-Jun19 30 mts (*n*=74)	**Improved Scenario** Jul19-Dec21 30 mts (*n*=52)	**All patients** (*n*=126)
**Recipients**			
Number	37	26	63
Age (median) [Q1-Q3]	40 [28 - 56]	47 [35 - 54]	44 [31 - 55]
Age category			
*18-39 y*	16 (43%)	9 (35%)	25 (40%)
*40-49 y*	3 (8%)	7 (27%)	10 (16%)
*50-59 y*	7 (19%)	6 (23%)	13 (20%)
*≥60 y*	11 (30%)	4 (15%)	15 (23%)
Sex			
*Male*	26 (70%)	17 (65%)	43 (68%)
*Female*	11 (30%)	9 (35%)	20 (32%)
Ethnicity			
*White non-Hispanic*	33 (89%)	25 (96%)	58 (92%)
*White Hispanic*	3 (8%)	0 (0%)	3 (5%)
*Black*	1 (3%)	1 (4%)	2 (3%)
*Other*	0	0	0
Pre-emptive status at referral	18 (49%)	11 (42%)	29 (46%)
Pre-emptive status at transplant	10 (27%)	8 (30%)	18 (28%)
Median recipient’s dialysis days at donor referral	169 [54 – 532]	175 [75 - 545]	112 [64 - 542]
Median recipient’s dialysis days at transplantation	345 [224 - 351]	258 [220 - 540]	407,5 [243 - 500]
Median recipients’ dialysis days while donor evaluation was underway	275 [262 - 416]	171 [107 - 223]	295,5 [176 - 317]
**Donors**			
Number	37	26	63
Age (median) [Q1-Q3]	57 [49 - 62]	57 [49 - 63]	57 [49 - 63]
Age category			
*18-39 y*	1 (3%)	1 (4%)	2 (3%)
*40-49 y*	8 (24%)	6 (23%)	14 (22%)
*50-59 y*	10 (25%)	8 (31%)	18 (29%)
*≥60 y*	18 (48%)	11 (42%)	29 (46%)
Donor-recipient relationship			
*Related*	23 (62%)	18 (69%)	41 (65%)
*Spousal*	11 (30%)	3 (12%)	14 (22%)
*Unrelated*	3 (8%)	5 (19%)	8 (13%)
Ethnicity			
*White non-Hispanic*	35 (94%)	25 (96%)	60 (95%)
*White Hispanic*	1 (3%)	0 (0%)	1 (2%)
*Black*	1 (3%)	1 (4%)	2 (3%)
*Other*	0 (0%)	0 (0%)	0 (0%)

### The base case: measurements and analysis

**Fig. 1:**
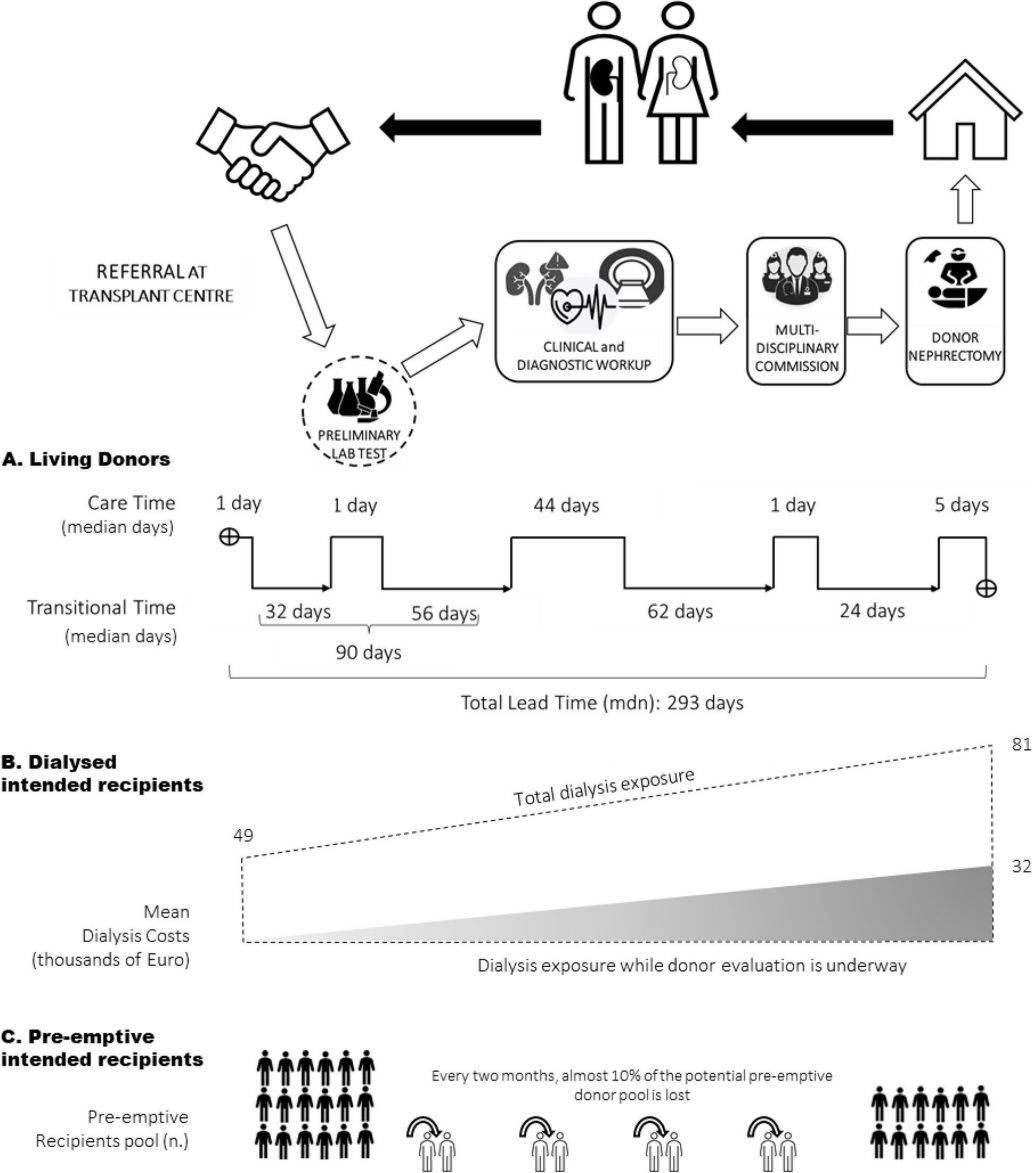
Expanded Value Stream Map. **A** Value stream mapping of living donor assessment process including care and transitional times. **B** Mean dialysis costs accrued by intended recipients: pre-transplantation dialysis, dialysis while donor evaluation is underway (grey area) and total dialysis at transplantation. **C** Variation of potential pre-emptive recipients’ pool while donor evaluation is underway

**Fig. 2:**
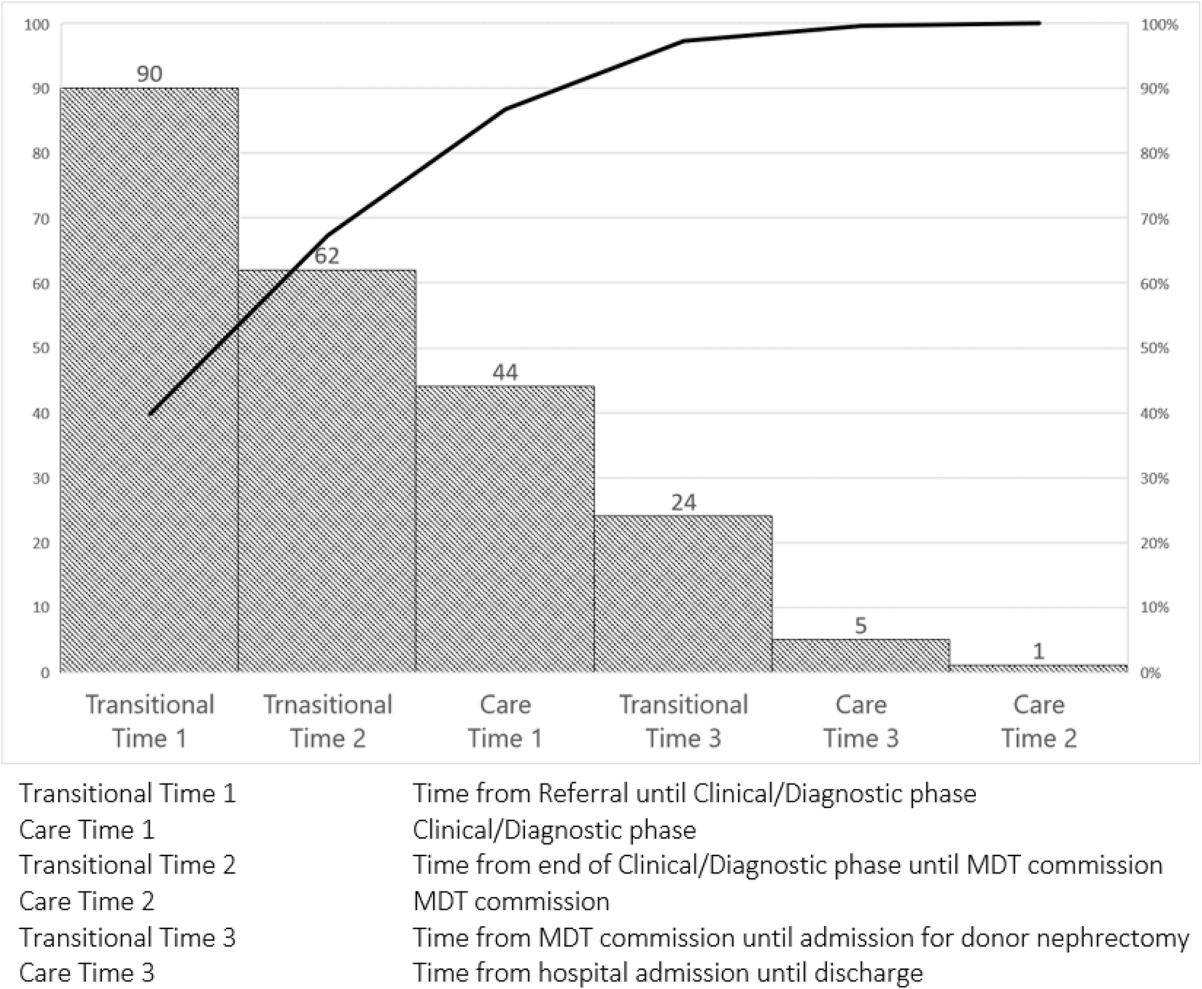
Pareto Chart. Pareto Chart displaying relative frequency of problems in order of defects (bars) and their impact on total lead time (line). The chart summarized problems according to highest priority

Between January 1, 2017 and June 30, 2019, 37 eligible donors were included. Median lead time is 293 days. However, only a median of 44 days is due to actual evaluation (care time). The vast majority of the process length is due to transitional time: 90 days to begin clinical-diagnostic workup, 62 days to reach commission approval and 24 days to schedule hospital admission and surgery (Fig. 1A). From the perspective of dialyzed waiting recipients, the process generates a median of 275 additional days of dialysis exposure. Cumulative dialysis time and costs accrued by intended recipients while donor evaluation is underway are 7912 days and 865k Euro, respectively. Mean dialysis expenditure for each waiting recipient is 81 thousand Euro, 40 percent of which while their donor evaluation was underway (Fig. 1B). Among the 18 pre-emptive recipients at referral, 8 (44%) need to begin dialysis while donor evaluation was underway. In other words, every two months the process dissipates 10% of the potential pre-emptive donor pool (Fig. 1C). Pareto Chart in Fig. 2 provides a visual representation of the burden of transitional time, The evaluation protocol is only the third most time-consuming phase while the first and second most time-consuming intervals are transitional intervals and together account for 65% of total lead time.

**Fig. 3:**
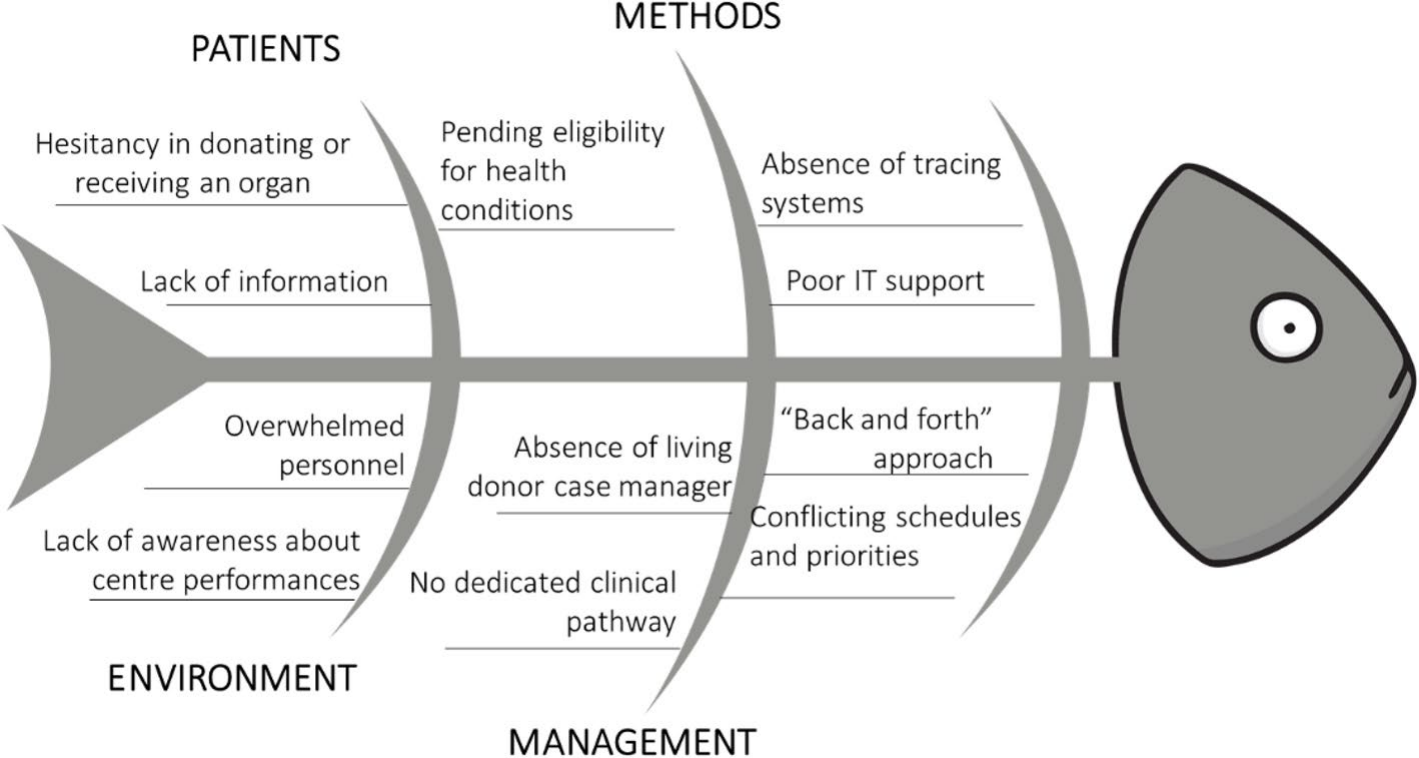
Fishbone analysis. Fishbone analysis illustrating main causes of flaws and bottlenecks current living donor assessment process

The qualitative analysis of this scenario revealed several potential explanations, summarized in Fig. 3. In particular, among all plausible causes, two of them were immediately perceived as critical ones:

#### Back-and-forth approach

According to current practice, LD must enter and exit our transplant centre at least six times: referral, submission of preliminary tests, at least three accesses for clinical-diagnostic evaluation (renal scintigraphy, cardiological assessment, and CT-angiography), and, eventually, surgery. A consequence of such fragmentation is, for instance, that by the time LDs submit their preliminary tests and the healthcare provides check them and schedule the first access to begin the workup, the process has already spoiled almost 3 months. An attempt to reduce such massive time loss was among our intervention’s priorities.

#### Poor process control

Among all stakeholders involved in the living transplant program, there was no one specifically appointed to control the process and orchestrate all players and actions toward the same goal. Poor control, in turn, might generate waste, fragmentation, and delays: belated assessment of submitted examinations; pending administrative, legal, and medical conditions that avert progression; patients having troubles in making and attending appointments. For instance, up to 25% of our examined population had duplicated exams or had to repeat them to update previous obsolete submissions. It is well acknowledged that the presence of a case manager who interfaces with all involved stakeholders, schedules and monitors the agenda, collects data, and supervises all steps of the process can facilitate progression of donor candidates [31–33].

**Fig. 4:**
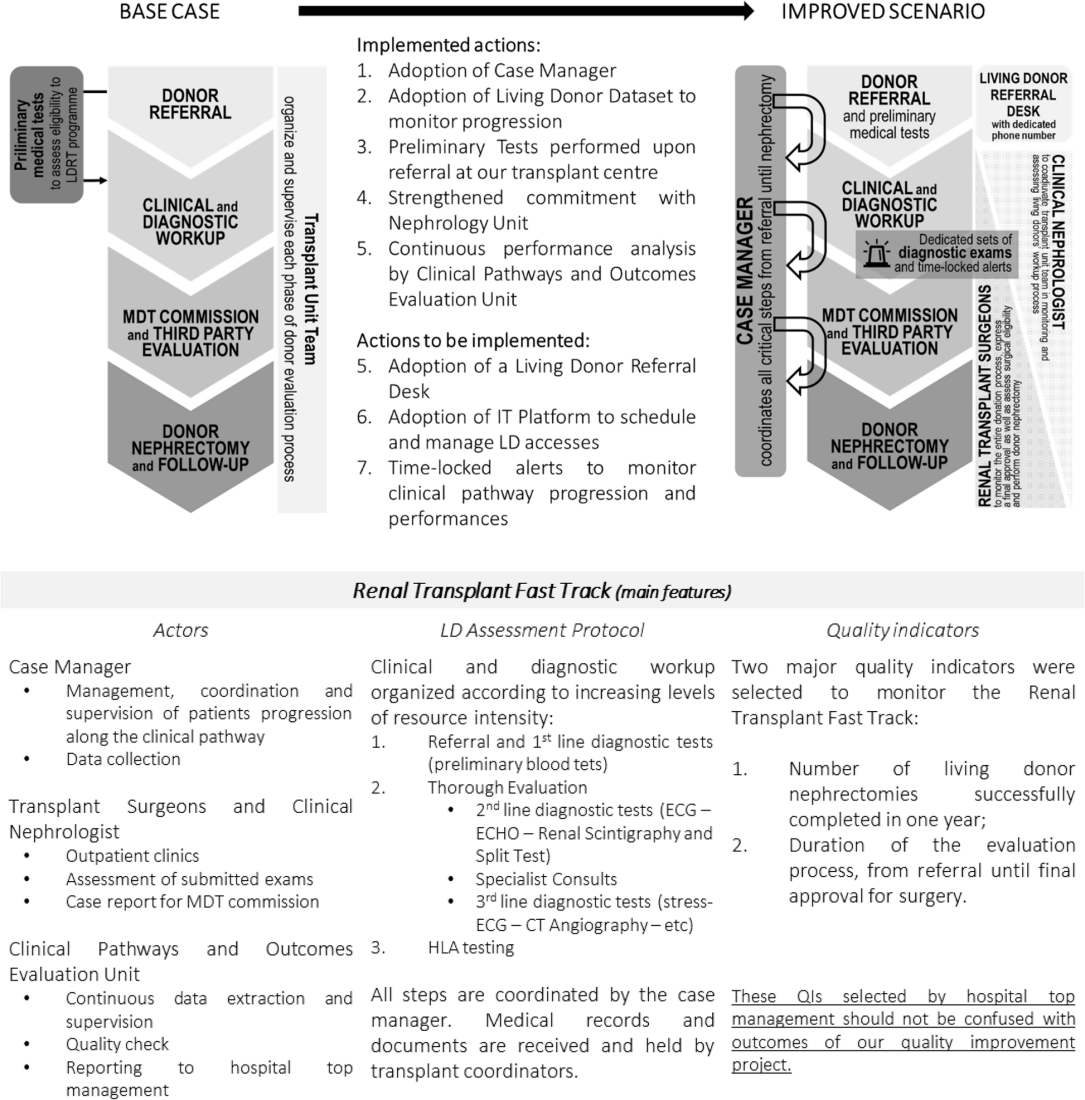
Base Case and Improved Scenario: critical features. (Top) Main steps in living donor assessment comparing Base Case and Improved Scenario with indications of implemented and to-be-implemented innovations. (Bottom) Definition of key roles and features of Renal Transplant Fast Track

This understanding of the process and its weaknesses suggested three critical actions. First, implementation of a case manager, a specialized nurse already involved in the living transplant program, who became responsible for coordinating all steps of the clinical pathway. Second, stronger commitment with the nephrology unit to promote living transplantation among ESRD patients and support its clinical pathway by having one nephrologist as part of the renal transplant team. Third, donor candidates were receiving the list of preliminary tests beforehand, to submit them immediately at referral. These interventions became the core of a new hospital protocol, called Renal Transplant Fast Track (Fig. 4), approved in October 2020. This protocol also reorganized diagnostic workup according to the increasing level of resource intensity, defined the role of team members and their tasks, and, most importantly, set quality indicators to monitor and adjust the process on a continuous basis.

### The improved scenario

**Fig. 5:**
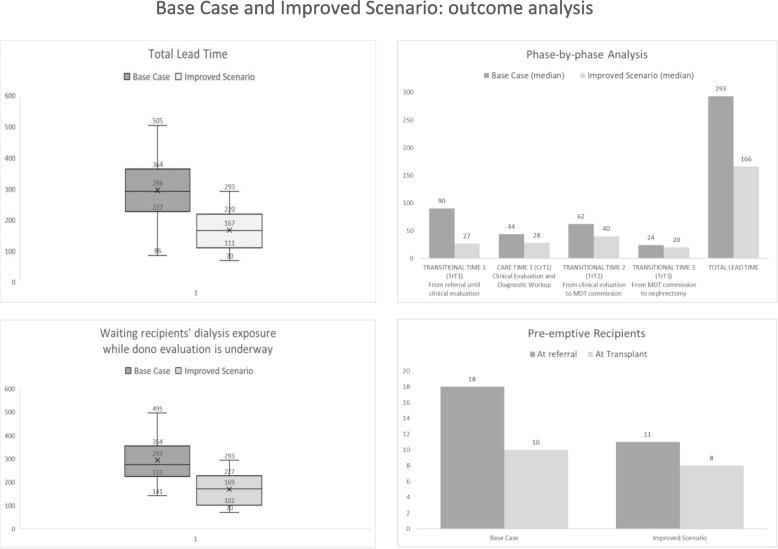
Outcome Table. Summary of outcomes: total lead time; phase-by-phase analysis; dialysis exposure while donor evaluation is underway; pre-emptive recipients. Comparison between base case and improved scenario

Between July 2019 and December 2021, 26 eligible donors were part of our prospective cohort. LD take a median of 27 days from referral to begin clinical evaluation which lasts a median of 28 days. Median total lead time is 166 days. Transitional phases absorb a total of 3883 cumulative days, a 52 percent reduction compared to base case performances. The clinical pathway generates a median of 171 days of additional dialysis exposure for waiting recipients, which corresponds to 3036 cumulative days and 332k Euro of dialysis expenditure for the healthcare system. Therefore, in the improved scenario there is a 45 percent reduction in median dialysis time and costs. Among 11 pre-emptive recipients at referral, 3 (27%) lost their pre-emptive status while donor evaluation was underway: it is a 38 percent reduction of the pre-emptive donor pool dispersion rate compared to the improved scenario. Results are summarized in Fig. 5.

## Discussion

LDRT is mentioned by all guidelines and recommendations as the most appropriate treatment for patients with ESRD. Nonetheless, cultural, psychological, and organizational barriers are responsible for its limited adoption. In this study we presented a QI project aimed at promoting LDRT by redesigning LD assessment pathway. Our intervention supports the idea that optimal process control is not only able to enhance efficiency but also to improve clinical outcomes and reduce healthcare costs, by shortening pre-transplantation dialysis exposure. Two further considerations are needed, however, to properly estimate the value of these achievements.

### Care time versus transitional time

This study was originally conceived to simulate the adoption of a one-day donor assessment model at our medical centre. This simulation, however, lead to very disappointing and counterintuitive results: even though all diagnostic steps were concluded in one day, time to transplantation was reduced only by 30 percent. Therefore, we decided to implement lean methodology to review and refine the entire process and not only the clinical and diagnostic evaluation. Value Stream Map in Fig. 1 indicates *care time* and *transitional time* to absorb about 35% and 65% of total lead time, respectively. In other words, more time is taken to move from one step to the next one than to complete each of those steps. Therefore, our efforts to conclude the diagnostic/clinical workup in one day could, at most, reduce *care time* but could not abbreviate *transitional time*. Although our study did not directly investigate the causes of transitional intervals, we can postulate some hypotheses. Release and approval of examinations and tests certainly absorb a fraction of this time as well as submission of a comprehensive medical record to MDT commission. Additional consultations due to candidates’ medical history or accidental findings should also be accounted for among causes of delay. Undoubtedly, intervals and pauses in any clinical pathway are not only unavoidable but even necessary to guarantee the accuracy and safety of the process: they might be considered *physiological breaks*. On the contrary, vigorous interventions are needed on *pathological breaks*, that disperse the clinical pathway’s rhythm and performance: lack of process standardization, the weak definition of roles and rules, poor data collection and awareness, inadequate engagement of other specialties, etc. These structure-related bottlenecks might be triggered or enhanced by donor-related ones, such as poor compliance with medical and organizational recommendations. A LD case manager is critical to limit such pathological breaks and to avoid subtracting time from the physiological intervals, that preserve patient’s safety, efficacy, and wellbeing.

### COVID-19: the unpredictable challenge

Italy was the first Western country hit by the COVID-19 pandemic in February 2020. As the burden of the pandemic enlarged, healthcare systems and facilities were entirely reorganized to allocate COVID-19 patients. In 2020, in Europe, the reduction of transplant activity ranged from 26.6% in the United Kingdom to 6.6% in Germany and organ donation activity from 27% in Portugal to 2% in Germany and similar data were registered worldwide [34]. In Italy, over 80% of transplant programmes reduced or stopped their evaluation protocols [35] and transplant activity registered a 10% contraction, which increases to 20% when looking at LDRT activity alone [16]. In addition, to advocate for donor, recipients and healthcare personnel safety, supplementary requirements were introduced for donor candidates [36–39]. In 2020 at our medical centre COVID-19 caused more than 3 thousand acute admissions and 760 ICU admission, which required hundreds of additional beds as well as the interruption of elective surgeries and outpatient clinics for months as well as deployment of more than 300 medical professionals to bear this additional burden [40]. Nonetheless, our LDRT programme registered performance improvement, that was maintained until the end of 2021. However, COVID-19 was largely responsible for the small number of living couples transplanted in 2020 – a 50% reduction when compared to 2019 – that can be explained by forced interruption of living transplant activity from the end of February to end of May and by patients’ hesitancy to access medical centres for risks of infection. This trend was reverted in 2021, when our medical centre registered the largest number of LD transplants ever acvhieved. Whether this increase in volumes has something to do with the Renal Transplant Fast Trask is beyond the means and scope of this article but, for sure, our model was able to absorb and react to COVID-19 shock.

Our study has some potential limitations. First of all, our analysis is simply a pre- and post- evaluation and, despite the magnitude of the results, we cannot claim any causal relationship between the intervention and the outcome. However, it is a valuable proof of concept to be further investigated in an experimental setting. Secondly, due to characteristics of our monitoring system, we were not able to record performance of all patients who referred at our centre but only those able to undergo transplantation successfully. This is an important bias, because quality is highly dependent on input volumes regardless for final outputs. A third limitation is strictly related to modest performance registered by the original evaluation protocol. Although follow-up cohort shows promising reduction in the evaluation time, it is also true that we started looking at the problem of a quite long evaluation time. Longer follow-up is therefore required to remove any possible confounding. Longer follow-up is also necessary to measure if and how much of our QI project translates into actual health for donors and recipients, such as graft and patient survival, rejection rates, graft function at 12 month and return to normal activity for donors: this will be the sequel of our study. A final limitation acknowledgment from the recipients’ side: most of them are already enlisted in deceased donor lists and eGFR at time of donor referral is not easy to obtain and, therefore, was not reported. This information would be very helpful to understand to what extend loss of pre-emptive status is due to process delays or belated donor referral at the living transplant centre.

## Conclusion

As clearly stated from the beginning, the aim of this study was to support the powerful role of clinical governance in designing and delivering assistance to patients. Without adopting any surgical, pharmacological or structural innovation , the new process provides quicker evaluation for LDs and higher chances for intended recipients to reach transplantation pre-emptively or with shorter pre-transplantation dialysis exposure, which, according to current scientific knowledge, translates into months and years of longer graft and patient survival [10, 41–43]. These same achievements reduce costs for healthcare systems and payers.

Fondazione Policlinico Gemelli is currently committed to refining and fully implement Renal Transplant Fast Track and to extend this approach also to other medical areas. Achievements for patients and healthcare system are immense. Value-oriented clinical governance is more than just filling organizational gaps with efficiency: it means quality and human care for patients and healthcare providers. Sharing this approach with other centres or other specialties might help healthcare systems – particularly universalistic ones – to survive the battle between growing health requests and progressively fading resources.

## Data Availability

The datasets used and/or analysed during the current study are available from the corresponding author on reasonable request.

## Abbreviations

LDRT: Living Donor Renal Transplant
QI: Quality Improvement
ESRD: End Stage Renal Disease
LD: Living Donor

## References

[1] Xie Y (2018). Analysis of the Global Burden of Disease study highlights the global, regional, and national trends of chronic kidney disease epidemiology from 1990 to 2016. Kidney Int.

[2] Bikbov B (2020). Global, regional, and national burden of chronic kidney disease, 1990–2017: a systematic analysis for the Global Burden of Disease Study 2017. Lancet.

[3] Abecassis M (2008). Kidney transplantation as primary therapy for end-stage renal disease: a National Kidney Foundation/Kidney Disease Outcomes Quality Initiative (NKF/KDOQITM) conference. Clin J Am Soc Nephrol.

[4] Tonelli M (2011). Systematic review: kidney transplantation compared with dialysis in clinically relevant outcomes. Am J Transplant..

[5] Cavallo MC (2014). Cost-effectiveness of kidney transplantation from DCD in Italy. Transplant Proc..

[6] Murtagh FEM (2007). Dialysis or not? A comparative survival study of patients over 75 years with chronic kidney disease stage 5. Nephrol Dial Transplant..

[7] Wolfe RA (1999). Comparison of mortality in all patients on dialysis, patients on dialysis awaiting transplantation, and recipients of a first cadaveric transplant. N Engl J Med..

[8] Meier-Kriesche H-U, Kaplan B (2002). Waiting time on dialysis as the strongest modifiable risk factor for renal transplant outcomes: a paired donor kidney analysis. Transplantation.

[9] Hart A (2019). OPTN/SRTR 2017 Annual Data Report: Kidney. Am J Transplant..

[10] Gill JS, Rose C, Joffres Y, Landsberg D, Gill J (2018). Variation in Dialysis Exposure Prior to Nonpreemptive Living Donor Kidney Transplantation in the United States and Its Association With Allograft Outcomes. Am J Kidney Dis.

[11] Schold JD, Meier-Kriesche H-U (2006). Which renal transplant candidates should accept marginal kidneys in exchange for a shorter waiting time on dialysis?. Clin J Am Soc Nephrol.

[12] Kasiske BL (2002). Preemptive Kidney Transplantation: The Advantage and the Advantaged. JASN.

[13] Jay CL, Dean PG, Helmick RA, Stegall MD (2016). Reassessing Preemptive Kidney Transplantation in the United States: Are we making progress?. Transplantation.

[14] van Dellen D (2021). Pre-emptive live donor kidney transplantation-moving barriers to opportunities: An ethical, legal and psychological aspects of organ transplantation view. World J Transplant.

[15] Axelrod DA (2018). An economic assessment of contemporary kidney transplant practice. Am J Transplant.

[16] Centro Nazionale Trapianti. Report 2020. Attività annuale della Rete Nazionale Trapianti. Available at: https://www.trapianti.salute.gov.it/imgs/C_17_cntPubblicazioni_415_allegato.pdf.

[17] Lentine KL (2017). KDIGO Clinical Practice Guideline on the Evaluation and Care of Living Kidney Donors. Transplantation.

[18] British Transplantation Society (2018). United Kingdom Guidelines for Living Donor Kidney Transplantation.

[19] Habbous S (2018). Initiating Maintenance Dialysis Before Living Kidney Donor Transplantation When a Donor Candidate Evaluation Is Well Underway. Transplantation.

[20] Habbous S (2018). Potential implications of a more timely living kidney donor evaluation. Am J Transplant..

[21] Alsharani M, Basonbul F, Yohanna S (2021). Low Rates of Preemptive Kidney Transplantation: A Root Cause Analysis to Identify Opportunities for Improvement. J Clin Med Res.

[22] Habbous S (2018). The Efficiency of Evaluating Candidates for Living Kidney Donation: A Scoping Review. Transplant Direct.

[23] Habbous S (2020). A RAND-Modified Delphi on Key Indicators to Measure the Efficiency of Living Kidney Donor Candidate Evaluations. Clin J Am Soc Nephrol.

[24] Attività Trapianto di Rene da Donatore Vivente dal 01 Gennaio 2001 al 31 Dicembre 2010. Available at: https://www.salute.gov.it/portale/documentazione/p6_2_2_1.jsp?lingua=italiano&id=1340.

[25] Graham JM, Courtney AE (2018). The Adoption of a One-Day Donor Assessment Model in a Living Kidney Donor Transplant Program: A Quality Improvement Project. Am J Kidney Dis..

[26] Régis TKO, Santos LC, Gohr CF (2019). A case-based methodology for lean implementation in hospital operations. J Health Organ Manag.

[27] Deblois S, Lepanto L (2016). Lean and Six Sigma in acute care: a systematic review of reviews. Int J Health Care Qual Assur.

[28] Kollberg B, Dahlgaard JJ, Brehmer P (2007). Measuring lean initiatives in health care services: issues and findings. Int J Product Perform Manag.

[29] Matthias O, Brown S (2016). Implementing operations strategy through Lean processes within health care – the example of NHS in the UK.

[30] Ogrinc G (2016). SQUIRE 2.0 (Standards for QUality Improvement Reporting Excellence): revised publication guidelines from a detailed consensus process. BMJ Qual Saf.

[31] Walsh MD, Barry M, Scott TE, Lamorte WW, Menzoian JO (2001). The role of a nurse case manager in implementing a critical pathway for infrainguinal bypass surgery. Jt Comm J Qual Improv.

[32] Siddique SM (2021). Interventions to Reduce Hospital Length of Stay in High-risk Populations: A Systematic Review. JAMA Network Open.

[33] Huntley AL, Johnson R, King A, Morris RW, Purdy S (2016). Does case management for patients with heart failure based in the community reduce unplanned hospital admissions? A systematic review and meta-analysis. BMJ Open.

[34] Concile of Europe. Newsletter Transplant, Preliminary Report 2020. Available at: https://www.trapianti.salute.gov.it/imgs/C_17_cntPubblicazioni_419_allegato.pdf.

[35] Vistoli F, et al. COVID-19 and kidney transplantation: an Italian Survey and Consensus. J Nephrol. 2020:1–14. 10.1007/s40620-020-00755-8 PMC7268183 32495231

[36] Jan MY (2021). A National Survey of Practice Patterns for Accepting Living Kidney Donors With Prior COVID-19. Kidney Int Rep.

[37] National Institute for Health and Care Excellence COVID 19 rapid guideline: renal transplantation: NICE Guideline [NG178]. 2020. Available at: https://www.nice.org.uk/guidance/ng178/chapter/3-Transplant-donors. 33378146

[38] American Society of Transplantation COVID-19 resources for transplant community. 2020. Available at: https://www.myast.org/covid-19-information.

[39] Frisullo G, De Belvis AG, Marca GD, Angioletti C, Calabresi P (2020). Stroke integrated care pathway during COVID-19 pandemic. Neurol Sci.

[40] Fondazione Policlinico Universitario A.Gemelli IRCSS; Università Cattolica del Sacro Cuore. Mission and impact Report 2020. https://www.policlinicogemelli.it/wp-content/uploads-shared/mir2020/#page=1.

[41] Kim HY (2019). Comparison of Clinical Outcomes Between Preemptive Transplant and Transplant After a Short Period of Dialysis in Living-Donor Kidney Transplantation: A Propensity-Score-Based Analysis. Ann Transplant.

[42] Prezelin-Reydit M (2019). Prolonged dialysis duration is associated with graft failure and mortality after kidney transplantation: results from the French transplant database. Nephrol Dial Transplant.

[43] Schold JD (2014). Association between kidney transplant center performance and the survival benefit of transplantation versus dialysis. Clin J Am Soc Nephrol.

